# Mastitis While Breastfeeding: Prevention, the Importance of Proper Treatment, and Potential Complications

**DOI:** 10.3390/jcm9082328

**Published:** 2020-07-22

**Authors:** Miri Pevzner, Arik Dahan

**Affiliations:** Department of Clinical Pharmacology, School of Pharmacy, Faculty of Health Sciences, Ben-Gurion University of the Negev, Beer-Sheva 84105, Israel; miripev@gmail.com

**Keywords:** mastitis, breastfeeding, lactation, breast infection, candida

## Abstract

Mastitis is an inflammation in the breast, which may involve a bacterial infection. Breast infection during breastfeeding is a common phenomenon that requires immediate and appropriate treatment. Without proper treatment, inflammation may lead to the cessation of breastfeeding. Another potential complication is the development of an abscess. Based on the nutritional and immunological value of breast milk, the recommendations of the American Academy of Pediatrics (AAP) and the World Health Organization (WHO) is exclusive breastfeeding up to 6 months of age, followed by continued breastfeeding as complementary foods are introduced, with continuation of breastfeeding for 1 year or longer as mutually desired by mother and infanRecent meta-analyses indicate breastfeeding protects against childhood infections, allows for a possible increase in intelligence, and for a reduction in overweight and diabetes. Breastfeeding is beneficial for nursing women as well. It is therefore important to help the mother overcome difficulties such as mastitis and to continue breastfeeding. The choice of a proper treatment and the provision of therapeutic instructions to the patient are crucial for a cure, for a successful duration of breastfeeding, and for preventing complications for mother and baby. In this article, we provide the latest clinical guidelines regarding drug treatment and supportive therapy in mastitis. We also discuss the latest guidelines of candida treatment, as candida infection may develop as a result of antibiotic therapy. Overall, improperly treated mastitis may cause the premature cessation of breastfeeding, and will cause suffering to both mother and baby; giving proper treatment and instructions to the mother are hence of the utmost importance.

## 1. Introduction

Mastitis is an inflammatory condition in the breast that may be accompanied by infection [1]. Breast inflammation during breastfeeding requires immediate and appropriate treatment. Without proper treatment, inflammation may cause the premature cessation of breastfeeding, which is considered the normative standard for infant feeding and nutrition. Based on the nutritional and immunological value of breast milk, the recommendations of the American Academy of Pediatrics (AAP) and the World Health Organization (WHO) are exclusive breastfeeding up to 6 months of age. After 6 month of life the recommendation is continued breastfeeding as complementary foods are introduced, with continuation of breastfeeding for 1 year or longer as mutually desired by mother and infant [2]. Recent meta-analyses indicate breastfeeding protects against childhood infections, allows for a possible increase in intelligence, and for a reduction in overweight and diabetes. For nursing women, breastfeeding gives protection against breast cancer, and it might also protect against ovarian cancer and type 2 diabetes [3]. In addition, improperly treated inflammation can develop into a breast abscess. Therefore, choosing the right treatment and providing information and therapeutic guidelines to the patient is of great importance. However, experience shows that women receive incorrect guidelines for the treatment of mastitis both in the aspect of the non-drug treatment guidelines and in adjusting the drug treatment. Given the great importance of the proper treatment of mastitis, it is important for physicians to be skilled in providing correct guidelines and choosing the right medication when needed.

In this article, we provide the latest clinical guidelines of drug treatment and supportive therapy in mastitis. We also discuss the latest guidelines of candida treatment, as candida infection may develop as a result of antibiotic therapy. Overall, improperly treated mastitis or candida infection may cause the premature cessation of breastfeeding, and will cause suffering to both mother and baby; giving proper treatment and instructions to the mother are hence of the utmost importance.

## 2. Definition, Diagnosis and Prevention

**Figure 1:**
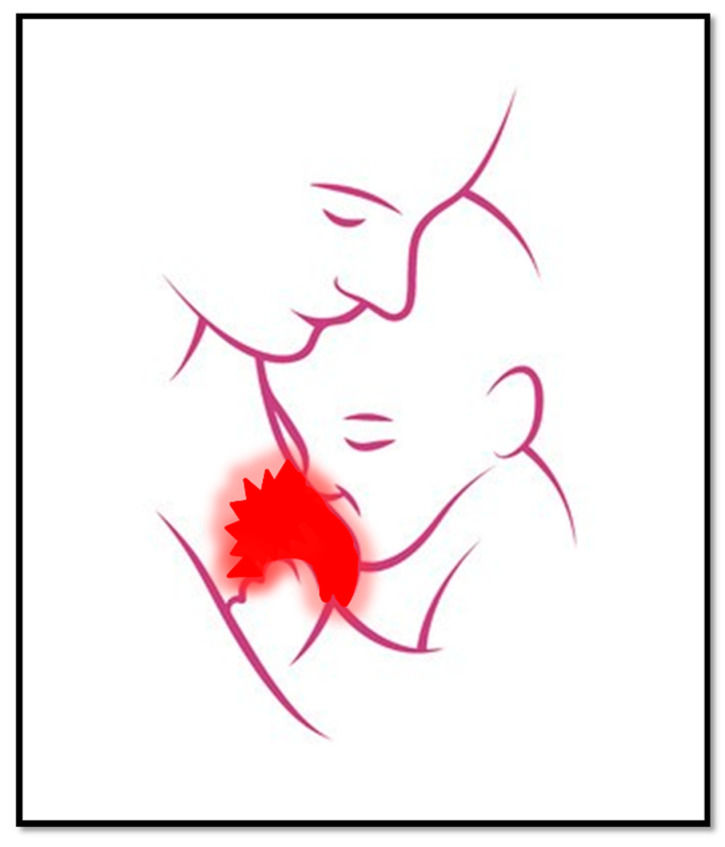
Illustration of mastitis while breastfeeding. Overall, improperly treated mastitis may cause the premature cessation of breastfeeding, and will cause suffering to both mother and baby; giving proper treatment and instructions to the mother are hence of utmost importance.

Breast inflammation may be due to a number of different etiologies, infectious or not, but most breast inflammation is expressed as a hard, swollen, and red breast area, accompanied by a fever above 38.5 °C, chills, and a bad general flu-like feeling (Figure 1). Many times there is a continuum, namely: stasis of breast milk develops into an inflammation without infection, which develops into infectious mastitis that may later develop into an abscess [4].

Prospective studies in women surveyed under the Academy of Breastfeeding Medicine protocol indicate that 3% to 20% of women suffer from mastitis while breastfeeding. The difference between the numbers is due to differences in the definition of mastitis and differences in the length of the follow-up period [4].

The main initiating factor of mastitis is milk stasis. Milk stasis leads to the blockage of a milk duct within the breast or in the opening of a milk duct. A blockage at the opening of a milk duct is manifested by the appearance of a sore white spot of about 1 mm on the nipple, known as a “bleb”, which is an epithelial layer or an oily material accumulation [5]. The usual treatment for a bleb is opening it with a sterile needle or rubbing the skin with fabric soaked in warm water after warming the nipple in hot water [5]. Steroidal ointments may also be effective [6].

The diagnosis of mastitis is made based on anamnesis and physical examination, and diagnostic procedures are not routinely needed or performed. WHO guidelines for sending a milk sample to the laboratory include the following: in case there is no response to antibiotic treatment within two days, in case of an allergy to the antibiotic treatment, in case of recurrent breast infections, in case of a hospital acquired infection, in cases requiring hospitalization, or in severe and unusual cases [1,4].

Breast milk is not sterile. Bacteria is present on the nipple, the skin of the breast, and even the milk ducts, and therefore the very presence of bacteria in the milk does not necessarily indicate mastitis [1,4].

Several factors may cause milk stasis, resulting in duct blockage and inflammation, including an attempt to increase the time between feeding or restricting the duration of breastfeeding, skipping feeding, incorrect latch of the baby to the nipple or ineffective suction, maternal or infant disease or birth defects, tongue tie, overproduction of milk, rapid and non-gradual weaning, pressure exerted on the breast by a bra, or nipple wound causing pain and thus delaying the next feeding [4]. Optimizing breastfeeding technique is likely to be beneficial to prevent mastitis. The mother should be aware of factors which may cause milk stasis and prevent them.

## 3. Treatment

### 3.1. Supportive Treatment

Effective milk removal: In cases of milk stasis at a deeper location in the breast tissue, the most important management step is frequent and effective milk removal. Mothers should be encouraged to breastfeed more frequently, starting on the affected breast. After feeding, expressing milk by hand or pump may contribute to good breast emptying, and thus contribute to healing; massage of the painful area toward the nipple helps to drain the breast properly [4,5].

In recent years, awareness of the importance of lymphatic massage in the treatment of mastitis has risen. To promote fluid drainage toward the axillary lymph nodes, the mother should massage the skin surface from the areola to the axilla [7].

There is no evidence of risk to the healthy, term infant for continuing breastfeeding from a mother with mastitis [4]. Women who are unable to continue breastfeeding should express the milk from breast by hand or pump, as the sudden cessation of breastfeeding leads to a risk of abscess development [4].

Sometimes infants refuse to breastfeed due to decreased milk production in the inflamed breast, a characteristic of mastitis, or due to a change in milk taste. Mastitis affects the biochemical composition of the milk, and as a result, the milk becomes saltier [8,9]. A woman who is unable to breastfeed the inflamed breast due to the baby’s refusal or for any other reason should pump or hand express the milk, as a sudden cessation of milk removal can cause the development of an abscess [1,4].

A hot compress or hot shower immediately before breastfeeding or suction can facilitate the release of milk from the breast. Cold compresses after breastfeeding or pumping and between breastfeeding will reduce any possible pain or edema [4,5].

In addition to effective milk removal, rest, nutrition, and drinking enough fluids are essential steps that can help the healing process [4,5].

### 3.2. Pharmacological Treatment

First, it is important to emphasize that supportive therapy should be continued concurrently with drug treatment. Medication alone is not enough.

Analgesics: Pain interferes with the milk ejection reflex, and therefore the mother should be encouraged to take analgesics. As ibuprofen has anti-inflammatory properties in addition to analgesia, it has an advantage over paracetamol. Ibuprofen at doses of up to 1.6 g per day is considered safe for breastfeeding [4].

Antibiotics: If symptoms do not improve within 12–24 h of starting treatment, antibiotic therapy should be started [4]. Table 1 summarizes the antibiotics that are commonly used in the treatment of mastitis.

The bacterium Staphylococcus aureus (*S. aureus*) is considered to be the major etiologic factor in bacterial mastitis, and therefore the relevant antibiotic types are effective against it. In recent years, there has been a rise in the United States in cases of infection with *S. aureus* resistant to methicillin (MRSA) in breast infections [4,5,10].

The usual duration of treatment is 10–14 days [4,5,10]. If there is no improvement within 48 h, a change in antibiotic should be considered while waiting for the results of the culture [4,5,10]. It is important to note that amoxicillin without clavulanate is not a suitable treatment option due to the high degree of resistance.

If the mastitis develops into an abscess, a combination of drainage and appropriate antibiotic therapy will be required to achieve healing. After drainage, breastfeeding on the side in which the abscess was present should continue with a posture where there is no direct contact between the baby’s mouth and the wound [4]. In case the baby cannot be positioned so that the mouth does not touch the wound, it is very important to take the milk out of the breast by pumping or expressing it manually. The continued removal of breast milk frequently after drainage will accelerate healing [10].

The recurrence of mastitis at the same site in the breast several times or the nonstandard onset of inflammation require evaluation to rule out tumor or other anomalies [4].

One of the possible consequences of the antibiotic therapy of mastitis may be the pathological growth of candida on the nipple. The main symptoms of candidiasis in the nipple include burning, shooting, stabbing, or deep cords pain, which occur during or after breastfeeding, and can also be felt between sessions [10]. Although candida-induced nipple area dermatosis is often characterized by a glossy pink nipple appearance with delicate or scaly nipples [11,12,13], the nipples and breast may also have a good appearance [4,10].

Newer research is disproving the causative agent as yeast, and implicating bacterial imbalance instead [6]. Recent literature does not support the existence of a candida infection deep in the breast; therefore, oral fluconazole therapy is irrelevant.

Topical antifungal preparations are recommended as the first line of treatment in breastfeeding [4,10,14,15]. Clotrimazole or miconazole should be applied to the nipple after each feed or every 3–4 h [14,15]. Steroid combination ointment can be considered in cases of pain and a clear inflammatory component [14,15,16].

Any excess cream may be gently wiped away before the next feeding [14]. The mother and the baby both should be treated for a week or longer [10,14,15,16,17]. Oral gel is not formulated for skin application, and is less effective in treating nipple candida than the cream [14].

In cases of open cracks on the nipples due to fungal infection, and where local antifungal therapy alone does not help, antifungal treatment may be combined with antibiotic mupirocin ointment [14,16,17].

Taking a culture is not usually necessary, but culturemay be taken if the diagnosis is unclear, if bacterial infection is suspected, or if there is no improvement after the initial treatment [15].

## 4. Conclusions

In conclusion, improperly treated mastitis may cause the premature cessation of breastfeeding, and will cause suffering to both mother and baby; giving proper treatment and instructions to the mother are hence of the utmost importance.

## Figures and Tables

**Table 1:** Antibiotics commonly used in the treatment of mastitis. Please note that amoxicillin without clavulanate is not a suitable treatment option due to high degree of resistance.

Antibiotic	Dosage	Notes
Cephalexin	500 mg × 4 times/day	Not suitable in case of allergy to penicillin with sensitivity to cephalosporins or with anaphylactic reaction to penicillin (severe allergy).
Amoxicillin-clavulanate	875 mg × 2 times/day	
Dicloxacillin	500 mg × 4 times/day	
Clindamycin	300 mg × 4 times/day	May be effective in the case of methicillin-resistant Staphylococcus aureus. An appropriate option in case of severe allergy to penicillin.
Trimethoprim-sulfamethoxazole	800–160 mg × 2 times/day	May be effective in case of methicillin-resistant Staphylococcus aureus. Avoid using if the baby is less than one months old, or if the baby is jaundiced, ill, premature or G6PD
